# Structure and Stability of the Spinach Aquaporin SoPIP2;1 in Detergent Micelles and Lipid Membranes

**DOI:** 10.1371/journal.pone.0014674

**Published:** 2011-02-14

**Authors:** Inés Plasencia, Sabeen Survery, Sania Ibragimova, Jesper S. Hansen, Per Kjellbom, Claus Helix-Nielsen, Urban Johanson, Ole G. Mouritsen

**Affiliations:** 1 Department of Physics and Chemistry, MEMPHYS-Center for Biomembrane Physics, University of Southern Denmark, Odense, Denmark; 2 Department of Biochemistry and Structural Biology, Center for Molecular Protein Science, Lund University, Lund, Sweden; 3 DTU Physics, Technical University of Denmark, Lyngby, Denmark; 4 DTU Nanotech, Technical University of Denmark, Lyngby, Denmark; 5 Aquaporin A/S, Copenhagen, Denmark; Hospital 12 Octubre Madrid, Spain

## Abstract

**Background:**

SoPIP2;1 constitutes one of the major integral proteins in spinach leaf plasma membranes and belongs to the aquaporin family. SoPIP2;1 is a highly permeable and selective water channel that has been successfully overexpressed and purified with high yields. In order to optimize reconstitution of the purified protein into biomimetic systems, we have here for the first time characterized the structural stability of SoPIP2;1.

**Methodology/Principal Finding:**

We have characterized the protein structural stability after purification and after reconstitution into detergent micelles and proteoliposomes using circular dichroism and fluorescence spectroscopy techniques. The structure of SoPIP2;1 was analyzed either with the protein solubilized with octyl-β-D-glucopyranoside (OG) or reconstituted into lipid membranes formed by *E. coli* lipids, diphytanoylphosphatidylcholine (DPhPC), or reconstituted into lipid membranes formed from mixtures of 1-palmitoyl-2-oleoyl-phosphatidylcholine (POPE), 1-palmitoyl-2oleoyl-phosphatidylethanolamine (POPE), 1-palmitoyl-2-oleoyl-phosphatidylserine (POPS), and ergosterol. Generally, SoPIP2;1 secondary structure was found to be predominantly α-helical in accordance with crystallographic data. The protein has a high thermal structural stability in detergent solutions, with an irreversible thermal unfolding occurring at a melting temperature of 58°C. Incorporation of the protein into lipid membranes increases the structural stability as evidenced by an increased melting temperature of up to 70°C.

**Conclusion/Significance:**

The results of this study provide insights into SoPIP2;1 stability in various host membranes and suggest suitable choices of detergent and lipid composition for reconstitution of SoPIP2;1 into biomimetic membranes for biotechnological applications.

## Introduction

MIPs (major intrinsic proteins) are found in eubacteria, archae, fungi, plants and animals [1]. According to substrate specificity, MIPs are mainly classified into AQPs (aquaporins - or water channels) if they are only permeable to water, or GLPs (aquaglyceroporins - or glycerol-uptake facilitators) if they also facilitate passive diffusion of small solutes such as glycerol or urea [2], [3]. In addition, a structural role in the formation of cell junctions has been described for some MIPs [4].

Most members of the aquaporin super family have molecular masses, ranging from 25 to 31 kDa. The three-dimensional structures of several MIPs have been determined [5]–[7], and the quaternary structures of the proteins reveal that they all form homotetramers where each monomer acts as a functional unit [2]. Based on sequence similarity, the functional unit of all members in this family are predicted to have six hydrophobic, membrane-spanning α-helices connected by five loops of variable length that delimit a polar channel with two wide vestibules and a narrow pore [8], [9]. Two of the connecting loops, namely B and E, interact with each other from opposite sides through two highly conserved NPA (Asn-Pro-Ala) motifs forming a seventh transmembrane region that contributes to the pore region [10]. Highly conserved residues that stabilize the structure are found in the helices, e.g. the transmembrane helix-helix packing motif GXXXG [11], as well as conserved polar and charged buried residues that have been proposed to form hydrogen bonds and ion pairs [12].

Plants encode a very large and diverse MIP family. They have been classified into at least five subfamilies in higher plants: plasma membranes intrinsic proteins (PIPs), tonoplast intrinsic proteins (TIPs), the small basic intrinsic proteins (SIPs), NOD26-like intrinsic proteins (NIPs), and the recently discovered X intrinsic protein (XIPs) [13], [14]. PIPs form the most highly conserved subfamily in plants and are further divided into two groups named PIP1 and PIP2 [15]. In addition to several single amino acid residue substitutions, PIP2s are characterized by a short N-terminal and a longer C-terminal relative to PIP1s [16]. Moreover, differences are found in the water transport activity in oocytes where PIP2s are more active than PIP1s [16]. It has been suggested that PIP2s are specific for water, whereas PIP1s have been reported to facilitate the transport of solutes such as glycerol, boric acid, urea, and carbon dioxide in addition to water [17]–[19].

The spinach (*Spinacia oleracea*) genome contains at least three PIP1 and four PIP2 genes [20]. Recently, a new nomenclature which reflects the phylogenetic classification of plants MIP genes and proteins has been adopted [14]. In accordance with this nomenclature, the spinach PIPs PM28A, PM28B and PM28C have been renamed SoPIP2;1, SoPIP1;1 and SoPIP1;2, respectively. SoPIP2;1 can be overexpressed and purified with high yields [21] and its structure has been solved with Angstrom resolution [5], [22]. However, the structural stability parameters of SoPIP2;1 have not been examined neither when solubilized by detergents nor after the protein reconstitution into membranes subsequent to the heterologous expression.

Due to the fact that the transport characteristics of SoPIP2;1 is well described (e.g. from protein reconstitution into oocytes and *E. coli* membranes [23]) it is a good candidate for being used in technological applications, and the high selectivity and water permeability of SoPIP2;1 makes it particularly interesting for a biomimetic water filtration technology. AQP mediated water transport is a prominent example of how Nature itself has developed an effective mechanism for purifying water, and many technologies based on biomimetic membrane transport is now attracting considerable interest (for a review see [24]). However, successful reconstitution and stabilization of functional proteins in biomimetic membranes depends on suitable choices of both detergent and host lipid membrane components.

Detergents are commonly used to solubilize membrane proteins, and many membrane proteins have been solubilized with various detergents without the loss of biological activity [25]. Sugar-based detergents and poly(oxyethylen)-based detergents are at presently the most commonly used. In the particular case of SoPIP2;1 different detergents like octyl-β-D-thioglucopyranide (OTG) and octyl-β-D-glucopyranoside (OG) have been used. We encountered problems with protein stability using OTG, presumably related to the low solubility of OTG at low temperatures, leading to aggregation of the protein under these conditions. Consequently we performed our work using OG micelles where no stability problems occurred with any of the preparations used in this work.

One of the major challenges in designing biomimetic systems based on integral membrane proteins is the reconstitution of the proteins into the membrane. A common strategy involves the incorporation of detergent solubilized proteins into vesicles followed by detergent extraction. The detergent-free vesicles are then fused with a receiving membrane. Typical lipid species for fusogenic vesicles are palmitoyl-oleoyl-phosphatidylethanolamine (POPE) -phosphatidylcholine (POPC) and -phosphatidylserine (POPS) lipids supplied with sterols e.g. ergosterol [26]. Other common lipids for biomimetic membranes are *E. coli* lipid extracts and diphytanoylphosphatidylcholine (DPhPC). In this study we examine SoPIP2;1 stability in four different membrane systems: *E. coli* lipids, DPhPC, POPE: POPC: POPS∶ergosterol and POPE: POPC mixtures.

In this paper we present results from an extended secondary and ternary structural characterization of the protein in detergent micelles and in lipid membranes using spectroscopic techniques. Specifically we study the thermal secondary structure stability of SoPIP2;1 when reconstituted in detergent micelles or in lipid membranes using a detailed analysis by Circular Dichroism spectroscopy. In addition we performed Emission Fluorescent spectroscopy analysis of the six tryptophan (Trp) amino acids present in the primary sequence of SoPIP2;1 in order to obtain information about the thermal ternary structure stability of the protein.

## Results

First we characterize the folding patterns for SoPIP2;1 in detergent and lipid. Then we characterized the thermal stability of SoPIP2;1 secondary and tertiary structure.

### SoPIP2;1 α-helical content in different systems

**Figure 1:**
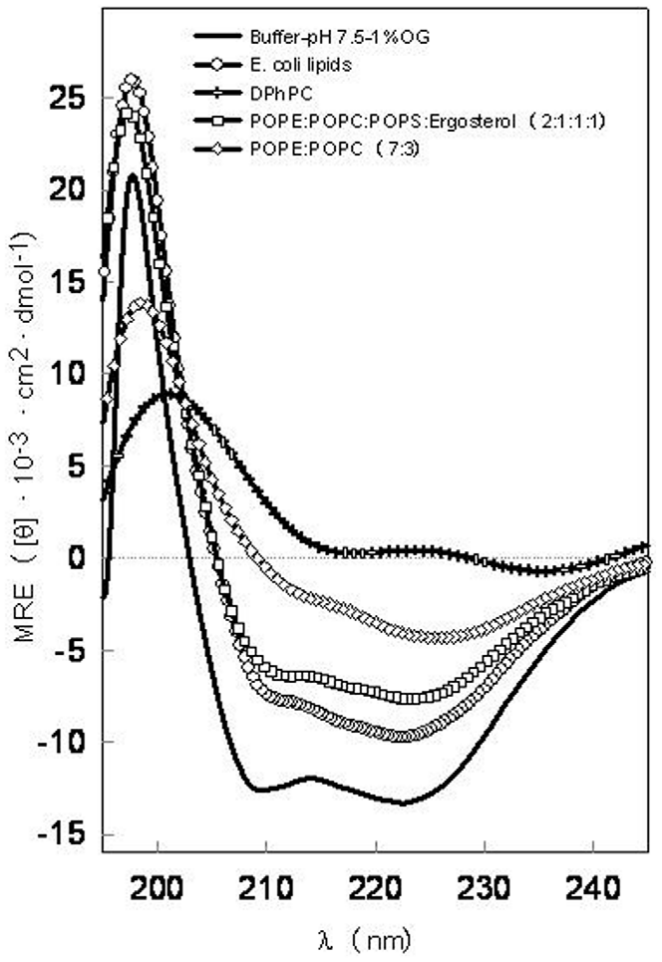
Far-UV CD spectra of SoPIP2;1. The measurements were obtained at 20°C in phosphate buffer, pH 7.5, NaCl 150 mM containing 1% OG and in different lipid membranes.

The far-UV CD spectra of SoPIP2;1 dissolved in PBS with 1% OG display the characteristics of a predominantly α-helical protein with negative bands around 209 nm and 222 nm (Figure 1, full line). The deconvolution analysis reported a α-helix content of 63% (Table 1). This value matches perfectly with the one obtained after the analysis of the PDB file 1Z98 from the X-ray diffraction data of this protein [22]. Although light scattering tend to flatten out the CD spectra obtained from protein in lipid vesicles making a direct comparison to micelles difficult, the characteristic α-helical structure for aquaporins is rather well preserved when the SoPIP2;1 is reconstituted in lipid complex membranes of *E. coli* lipids (Figure 1 and Table 1). This is consistent with previous results demonstrating protein activity (water flux) regardless if SoPIP2;1 is purified in OTG [21] or OG (unpublished results, J.S. Hansen). Other aquaporins have also been successfully reconstituted in *E. coli* lipid membranes [27], [28].

**Table 1:** Summary of the SoPIP2;1 secondary structure results.

	%
	α-Helix	β-sheet	Turns	Random
**OG micelles**	63	20	3	15
***E. coli*** ** lipid**	61	17	7	16
**POPE∶POPC∶POPS∶Ergosterol**	48	25	6	18
**POPE∶POPC**	43	27	9	21
**DPhPC**	42	30	7	21

We then tested whether SoPIP2;1 could be reconstituted in DPhPC membranes as DPhPC is generally recognized as forming stable black lipid membranes [29]–[31], making this lipid, in principle, suitable for being used in the design of water-filtration systems. However our CD analysis of the SoPIP2;1-DPhPC mixture suggested that this lipid does not preserve the structure of this protein. Although spectra could be obtained (see Figure 1) reproducibility was low for SoPIP2;1-DPhPC mixtures. White precipitate particles were commonly observed during the reconstitution process which may be directly related to the apparition of denatured protein aggregates. The aggregation of the protein in DPhPC could be related to the difficulties encountered in forming liposomes even when extrusion methods were used. Also dynamic light scattering analysis of the DPhPC suspension reported very broad structural features. Specimens with diameters ranging from 51 to 2669 nm (range of the diameters taken from the calculated Gaussian distribution of the sizes between 10% and 90% of the total distribution) were found in the same sample. The polydispersity index was 0.308, confirming a high liposome structural heterogeneity. These results suggest that this lipid does not provide a good membrane environment for a successful and stable SoPIP2;1 membrane incorporation. Hence, we exclude DPhPC bilayers as a host lipid membrane for SoPIP2;1.

We then examined POPE∶POPC∶POPS∶Ergosterol (2∶1∶1∶1 molar ratio) as this mixture has been shown to be as a good fusogenic lipid mixture [32] applicable for the further incorporation of aquaporins into industrial membrane water-filtration systems. The CD spectrum from SoPIP2;1/POPE∶POPC∶POPS∶Ergosterol mixture does not look very different from the one obtained when the protein is reconstituted into *E. coli* lipid membranes (Figure 1). However, the α-helical content reported after the deconvolution analysis is lower (Table 1) and associated with an increase in the β-sheet content and the other analyzed structures. The spectra obtained with another suitable fusogenic lipid mixture, POPE∶POPC (7∶3 molar ratio), showed even less helical-like spectra although the deconvolution results report values similar to those obtained with the more complex fusogenic mixture (Figure 1 and Table 1).

Taken together we conclude that the best lipid system identified for the reconstitution of SoPIP2;1 is *E. coli* lipid membranes, followed by POPE∶POPC∶POPS∶Ergosterol and POPE∶POPC. In contrast DPhPC was found not to be suitable for the SoPIP2;1 membrane reconstitution.

### Thermal stability of SoPIP2;1 secondary structure

**Figure 2:**
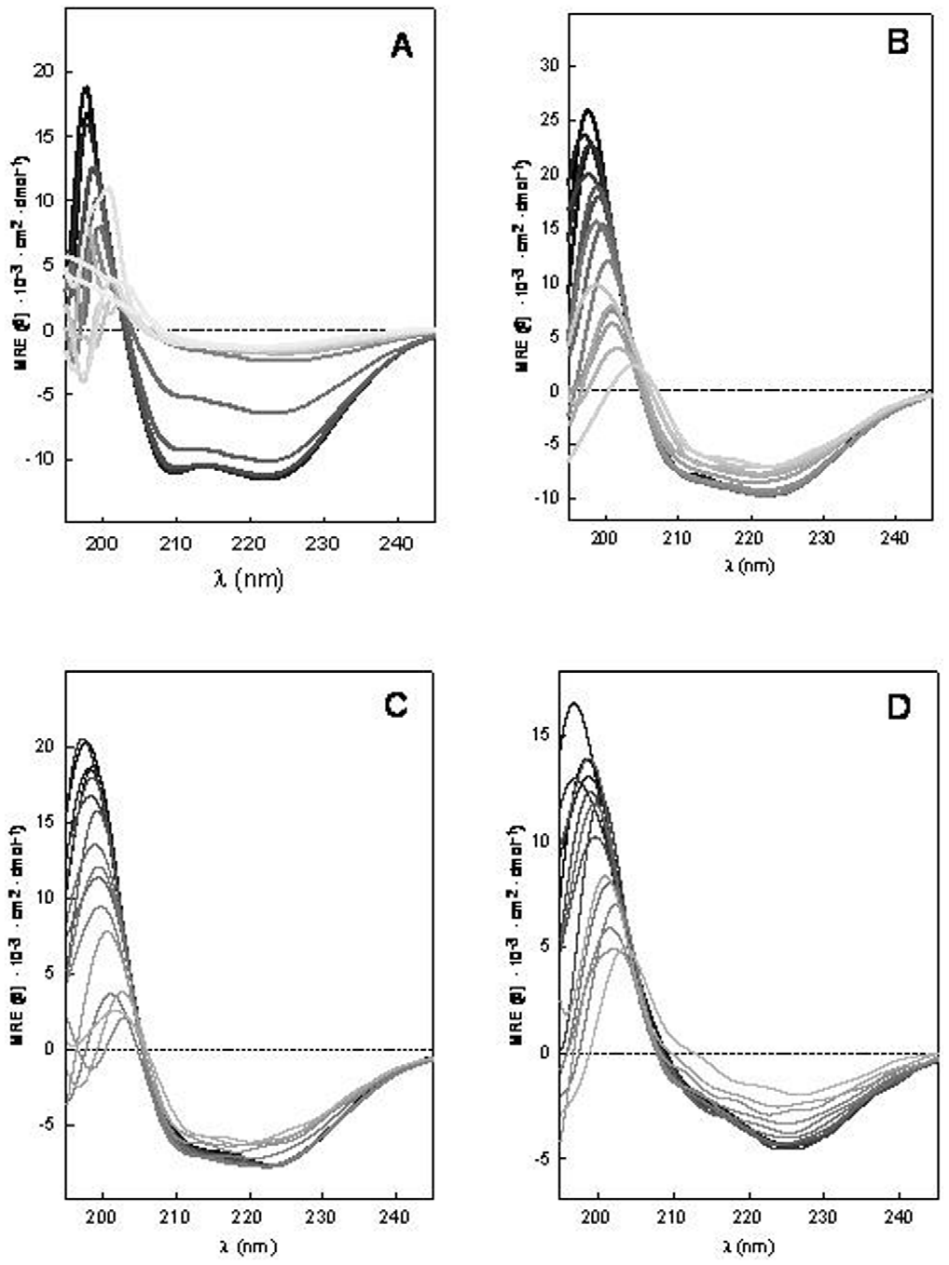
Thermal unfolding of the secondary structure of SoPIP2;1. Protein was reconstituted in A) OG micelles, B) *E. coli* lipid membranes, C) POPE∶POPC∶POPS∶Ergosterol membranes and D) POPE∶POPC membranes. Temperatures are represented by the grayscale colors from black, 20°C to very light gray, 95°C.

One method to characterize the unfolding process of a protein is to study the effect of temperature changes on its structure. This provides important information about the conformational stability of protein. The thermal stability of SoPIP2;1 was found to differ between detergent micelles and the different lipid species studied here, as evidenced by CD spectroscopy (Figure 2). The changes in the secondary structure spectra associated with temperature increase was found to be larger for the protein reconstituted into detergent micelles (Figure 2, A) compared to *E. coli* lipids (Figure 2, B), the POPE∶POPC∶POPS∶Ergosterol mixture (Figure 2, C) and the POPE∶POPC mixture (Figure 2, D).

**Figure 3:**
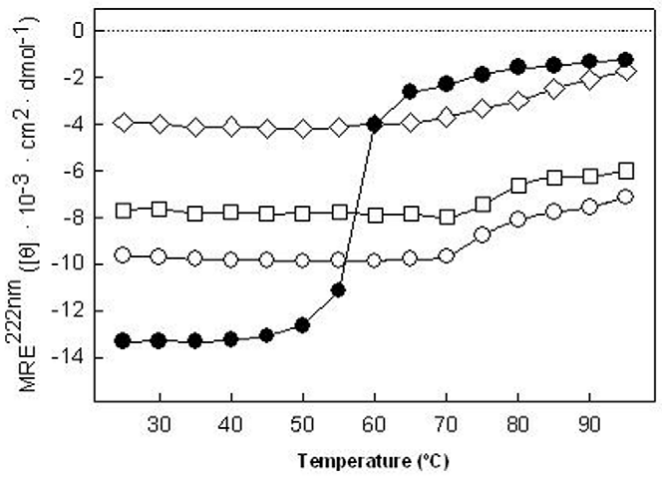
MRE at 222 nm as a function of temperature. SoPIP2;1 in OG micelles (•), *E. coli* lipid membranes (□), POPE∶POPC∶POPS∶Ergosterol ( o) and POPE∶POPC (◊).

A summary of the results can be found in Figure 3 for the thermal stability of the secondary structure where the Mean residue ellipticity (MRE) at 222 nm were plotted as a function of the temperature for all systems studied. The different MRE values at 20°C (initial state) demonstrate that the protein is structured or interacting in different ways with the different detergents or lipids used. Therefore it can be assumed that the protein acquires different structural initial states as quantified by the deconvolution analyses in the different environments tested (Table 1). The SoPIP2;1 structure is more sensitive to the temperature changes when it is reconstituted into detergent micelles than when it is reconstituted into lipid membrane systems. Although the unfolding transition is not reversible, the transition midpoint can be used to quantify the thermal stability of the protein [33].

**Figure 4:**
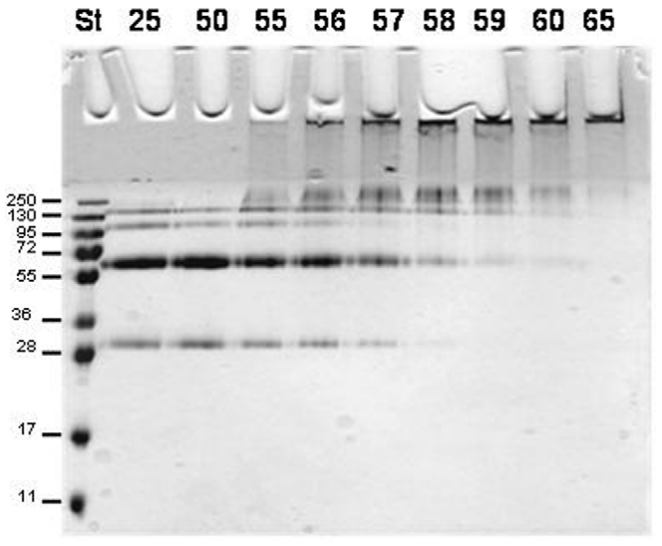
Thermal denaturation of SoPIP2;1. It can be visualized by SDS-PAGE since large aggregates are formed that cannot enter the gel. After incubation at room temperature the normal bands corresponding to monomeric, dimeric, trimeric and tetrameric SoPIP2;1 are evident. No aggregates were found at 50°C whereas at the melting point, 58°C, as determined by CD, part of the protein was aggregated. At 65°C the aggregation was complete. The samples were incubated at the indicated temperatures (25–65°C) for 10 minutes before sample loading buffer was added followed by 10 minutes incubation at room temperature. St, standard with indicated Mw in kDa in the left side of the gel.

The variation of MRE 222 nm with increasing temperature shows that the protein in detergent micelles solution has a melting temperature around 58°C. The decreasing temperature ramp measurement failed to report the same initial state (data not shown), indicating an irreversible unfolding of the protein under these conditions. A white precipitate in the sample after incubation at the melting temperature was observed probably caused by the presence of unfolded protein aggregates that precipitated at the bottom of the sample cuvette. Thus the structural stability of the protein can be maintained up to 50°C. The melting temperature value around 58°C was also confirmed by SDS-PAGE (Figure 4). Presence of monomers and dimer bands can be observed at temperatures below 58°C but these bands disappear at higher temperatures and aggregated protein was retained in the well. Bands corresponding to complexes larger than the tetrameric protein are visible between 55 and 60°C.

Protein reconstitution in complex membranes lipid mixtures (*E. coli* or POPE∶POPC∶POPS∶Ergosterol) increases the stability of SoPIP2;1 against temperature changes in the system. The spectra of the protein reconstituted in these membranes exhibited a different behavior in response to the temperature increase compared to the protein reconstituted in detergent-micelles (Figure 2). In this case of reconstitution in membrane lipid mixtures, only minor changes were observed in the spectra and appeared first above 70°C. This is clearly evident in the MRE 222 nm value representation (Figure 3).

### Thermal stability of SoPIP2;1 tertiary structure

**Figure 5:**
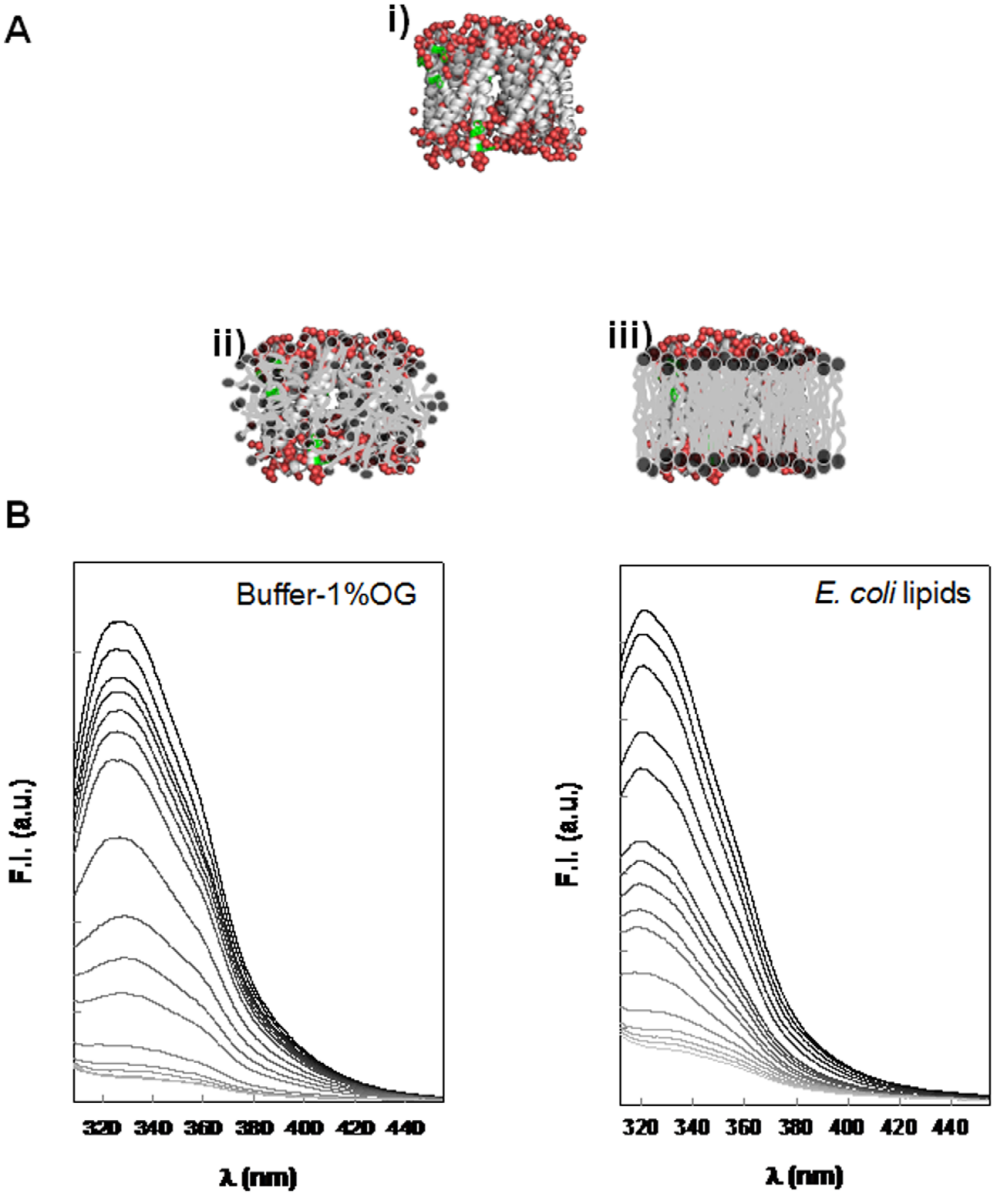
Fluorescence spectroscopy analysis of the protein ternary structure behavior. A) Cartoons illustrating the Trp residues (green) position in the protein ternary structure SoPIP2;1. The structure derives from the PDB file 1Z98 [22] using VMD software is shown in i [50], an illustration of the protein position in detergent micelles in ii and in lipid bilayers in iii. B) Thermal unfolding of SoPIP2;1 monitored by tryptophan fluorescence. To the left, the protein is reconstituted into OG micelles and to the right the protein is reconstituted into *E. coli* lipid membranes. Temperatures are represented by the grayscale colors from black, 20°C to very light gray, 95°C.

**Figure 6:**
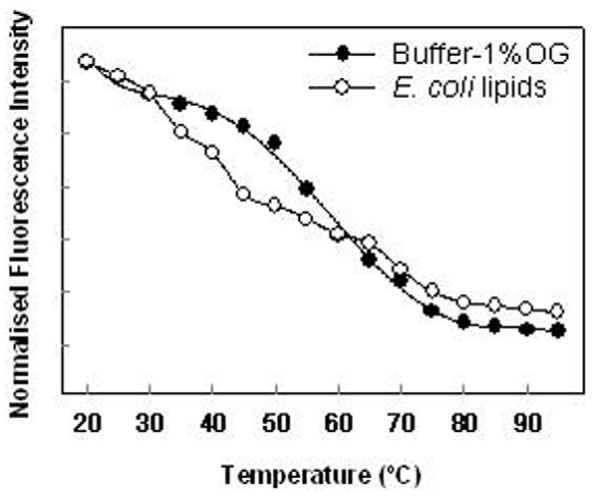
Fluorescence intensity at the maximum of the emission spectra as a function of temperature. The protein reconstituted in detergent micelles show one principal transitions in comparison with the protein reconstituted in lipid membranes where at least three different transitions can be observed. Fluorescence intensity values are normalized to the higher intensity value observed with the protein in micelles suspension at 20°C.

SoPIP2;1 contains six tryptophan (Trp) residues in the primary sequence. These residues can be used as intrinsic fluorophores for analyzing the protein ternary structure (see the cartoon in Figure 5A) as the Trp fluorescence emission spectrum is sensitive to both the polarity and the dynamics of the environment surrounding the aromatic side chain. Therefore, the variation in the Trp fluorescence emission spectra reports changes in tertiary structure of the protein. Thus the Trp is generally blue-shifted from 350 nm in environments of low polarity such as the hydrophobic interior of a protein or in a lipid membrane environment and Trp fluorescence in proteins has been classified into five classes by Reshetnyak et al. [34]. According to this classification, the dominant fluorescence around 329 nm corresponds to a class of Trp side chains that are in a relatively non-polar environment and H-bonded in an 2∶1 exciplex that fluoresces at 331 nm [34]. Inspection of the three-dimensional structure of SoPIP2;1 indicated that the Trps of this protein are positioned close to the surface of the protein (see cartoon i in Figure 5A) [22]. SoPIP2;1 reconstituted into detergent-micelles exhibited a maximum fluorescence at 330 nm indicating that the region of the protein where the Trps are located is positioned at the edge of the detergent micelles thereby facilitating the contacts between Trps and water molecules (see cartoon ii in Figure 5A and experimental results in Figure 5B). The easier accessibility of the water to the Trp environment is due to the loosely packed hydrophobic tails of the detergents interacting with the protein in detergent-micelles. A shift to lower wavelength values was always observed when the protein was reconstituted into lipid membranes (representative spectra showed with the *E. coli* membranes in Figure 5B). It demonstrates that the lipid membrane environment offers a more extensive hydrophobic surface for interaction with the transmembrane protein (cartoon iii in figure 5A). It is also consistent with the higher temperature stability exhibited by the protein in the membrane systems. Thermal stability of the protein was also monitored by following the Trp fluorescence vs. increasing temperature. The Trp fluorescence was quenched at higher temperatures while the wavelength at which maximum emission took place was not affected significantly (Figure 5B and 6).

For transmembrane proteins two components are generally contributing to a higher water access to the Trp location. One is the fluidity of the micelles or membranes and the other is the progressive unfolding of the protein. In case of detergent micelles, the effect of possible micelle fluidity is not very high. The protein reconstituted into micelle solution exhibited a very similar profile to the protein unfolding analyzed by CD. In this situation the principal transition occur around 58°C (Figure 6). When the protein is reconstituted in lipid membranes the profile is more complex with more than one transition indicating the contribution of both components to the final state (Figure 6). The first transition may reflect a change in the lipids that allows water to access the Trp environment without any structural change in the protein. In any case the steeper slope, likely to be related to the protein unfolding, occurs around 70°C correlating well with the data obtained by CD spectroscopy.

## Discussion

The structural stability of the protein is important as a precedent for good functionality after reconstitution. Here we used CD spectroscopy as an effective and fast tool for testing the structural properties of SoPIP2;1 and its stability in different environments. CD spectroscopy has already been used with many other proteins and it has been used for determining the melting temperature of proteins [33], [35], [36].

A high-resolution X-ray structure (PDB data 1Z98) has been published for untagged SoPIP2;1 [22], but the structural stability properties of SoPIP2;1 have never before been studied after overexpression and reconstitution into different membrane or membrane-like environments. A helical content of 60% was reported in the 1Z98 SoPIP2;1 crystallographic structure. However, the helical content determination is not complete as 1Z98 does not report the first 23 and the last 7 residues of the protein. Our CD measurements of His-tagged SoPIP2;1 in OG micelles, the same detergent also used to solubilize the protein for crystalization, showed a 63% helical content, a value that matches well with the high resolution structure of SoPIP2;1 demonstrating that CD spectroscopy is a very useful tool for testing the secondary structure stability of the protein reconstituted in different systems.

Regarding the tetrameric quaternary structure of SoPIP2;1, two mechanisms could explain the results shown in Figure 3: unfolding or dissociation of the tetrameric protein complex. The moderate change in MRE as function of temperature observed when the protein is reconstituted in phospholipid membranes could be related to protein unfolding in a small protein population. Considering that the CD results are giving an average value of the folding pattern this would imply that most of the protein is correctly folded. However, it is also possible that the change in MRE is due to dissociation of the aquaporin tetrameric complexes. In that case also monomers and dimers may occur in the membrane at higher temperatures giving rise to the changes in MRE as observed.

The information obtained by CD was also validated with Trp fluorescence emission. These measurements provided information about the protein tertiary structure and incorporation into the membrane. Our results revealed that POPE∶POPC∶POPS∶Ergosterol, POPE∶POPC and *E. coli* lipids all are appropriate lipid systems for reconstitution of SoPIP2;1 as they supported structural and thermal stability of the protein. The results from the latter system is consistent with the work by Karlsson et al. [21] using stop-flow measurements demonstrating water transport functionality for SoPIP2;1 in *E. coli* lipids. In contrast DPhPC does not provide a suitable host lipid membrane for SoPIP2;1.

It have been suggested that aquaporins exhibit greater stability [37]–[42] compared to the structurally closely related glycerol facilitators (aquaglyceroporins) [28], [43]–[45]. Galka and collaborators [36] showed using SDS-PAGE electrophoresis that *E. coli* GlpF aquaglyceroporin has an unfolding temperature of the tetramer around 60°C in detergent solutions of dodecyl β-D-maltoside (DDM). The same temperature was obtained for the tertiary structure monitored by Trp fluorescence [36]. When these authors studied the secondary thermal stability by far-UV CD spectroscopy they observed a two-state unfolding transition occurring around 70°C for GlpF in DDM. When GlpF was reconstituted into lysomyristoylphophatidylcholine (LMPC) micelles, the tetramer unfolded around 80°C and the thermal stability of the secondary structure was reported to have a transition temperature of 87°C. Compared with our results obtained for SoPIP2;1, GlpF in LMPC micelles apparently has a higher stability than SoPIP2;1 in OG micelles and phospholipid bilayers. On the other hand SoPIP2;1 reconstituted into the bilayer-forming phospholipid mixtures tested here shows a similar melting temperature as GlpF in DMM. Unfortunately a direct comparison is not possible as we do not have the same GlpF/lipid systems to compare with.

In conclusion we have shown that SoPIP2;1 can exist as a stable folded protein in OG detergent micelles solutions and that the protein can be transferred from detergent micelles solutions and reconstituted into selected phospholipid membranes preserving its structural characteristics. It is likely that more suitable reconstitution systems exist for SoPIP2;1 than those studied in the present work. In order to efficiently test a range of systems, new methods are called for. Presently we are working on developing a new microscopic method that will allow us to test at the same time the incorporation and distribution of the protein in different membrane systems and evaluate the yield of the incorporation and characterize the protein functionality.

## Materials and Methods

*E. coli* lipids total extract, 1,2-Diphytanoyl-sn-Glycero-3-Phosphocholine (DPhPC), 1-palmitoyl-2-oleoyl-*sn*-glycero-3-phosphoethanolamine (POPE), 1-palmitoyl-2-oleoyl-*sn*-glycero-3-phosphocholine (POPC) and 1-palmitoyl-2-oleoyl-*sn*-glycero-3-phospho-L-serine (sodium salt) (POPS) were purchased from Avanti Polar Lipids, Inc. (Alabaster, AL). Octyl-β-D-glucopyranoside (OG) was purchased from Anatrace (Maumee, OH) and Sigma-Aldrich (Brondby, Denmark). Ergosterol and all the other chemicals were obtained from Sigma-Aldrich (Brondby, Denmark).

### Heterologous protein overexpression

Functional SoPIP2;1 was overproduced in the methylotrophic yeast *Pichia pastoris* as His-tagged protein with a myc epitope [21]. The fusion protein has 303 amino acid and a molecular weight (Mw) of 32512 Da. The strain was grown in a 3 L fermentor typically resulting in 230 g wet cells/L culture after 24 h of methanol induction. Urea/NaOH washed membranes were prepared as described previously [21] and SoPIP2;1 was solubilised in 5% OG. Solubilized His-tagged material was purified using Ni-affinity chromatography as previously published [21]. The eluted protein was concentrated using a VivaSpin 20 concentrator (cutoff MW 10 kDa, VivaScience) and the buffer was then changed to 10 mM phosphate buffer, pH 7.5, NaCl 150 mM (PBS) supplemented with 10% glycerol and 1% OG using a PD-10 column resulting in a final concentration of 10–15 mg/ml as determined by Bearden [46].

### Electrophoresis

Pure protein (5 µg) in 20 µL of phosphate buffer (10 mM potassium phosphate, 150 mM NaCl, 1% OG and 10% glycerol pH 7.5) was incubated for 10 minutes at different temperatures (25°C–65°C). After incubation, sample loading buffer (125 mM Tris-HCl pH 6.8, 20% glycerol, 4% SDS, 10% (v/v) β-mercaptoethanol 0.1% bromophenol blue) was added to the protein and further incubated for 10 minutes at room temperature. SDS- PAGE (12%) of the sample was performed as described by Laemmli [47]. Protein was visualized by staining the gel with Coomassie brilliant blue R250.

### Dynamic Light Scattering (DLS)

Multilamellar 4 mg/ml DPhPC vesicles were formed by evaporation of the chloroform solvent by nitrogen gas and drying 30 minutes in the dessicator, followed by resolvation of the lipid film in sterile filtered 0.2 M KCl in double distilled water. The vesicle solution was extruded 11 times through a 100 nm polycarbonate filter. DLS size distribution analyses of 0.1 mg/ml DPhPC vesicles in 0.2 M KCl at 25°C were performed with a Malvern Zetasizer NanoZS instrument courtesy of LiPlasome Pharma A/S. Standard cuvettes (67.740 from Hounisen, Denmark) were used. 3 measurements of 13 runs each were taken and averaged. The analysis was carried out with the software program DTS 5.10, using an in-built general purpose analysis model.

### Liposome and Protoliposome reconstitution

Purified SoPIP2;1 was reconstituted into vesicles by mixing with *E. coli* lipids total extract or DPhPC or POPE∶POPC∶POPS∶Ergosterol (2∶1∶1∶1 mol ratio) (complex fusogenic mixture) or POPE∶POPC (7∶3 mol ratio) (binary fusogenic mixture) solubilized in 1% OG at a lipid-to-protein molar ratio (LPR) of 200 in phosphate buffer 10 mM, NaCl 150 mM, pH 7.5 at a final 0.1 mg/mL concentration of protein. The mixture was dialyzed in Slide-A-Lyzer® Dialysis Cassettes from Thermo Scientific, Pierce Biotechnology, (Rockford, IL) with a molecular cut-off of 10,000 Mw at room temperature for 4 days with two buffer changes per day. Control vesicles were made in the same manner without protein.

### Circular Dichroism (CD) and Fluorescence

CD spectra were acquired with a Jasco 815 spectrometer (Jasco UK, Essex, UK). The sample temperature was controlled by a built-in Peltier device. The protein or lipid-protein solutions were placed in a quartz cuvette with a 0.1 cm path length and the spectra were collected at 20 nm/min between 250–190 nm with a response time of 0.25 s and a data pitch of 0.1 nm. Baselines were collected in the same manner and spectra were baseline corrected. CD spectra for samples without protein, i.e. buffer or buffer with detergent and lipid did not exhibit ellipticity. Mean residue ellipticity (MRE) ([θ]×10^−3^ deg cm^2^ dmol^−1^) as calculated using the equation [θ]_M_ = Mθ/10·l·c·n, where M is 32512 g/mol, θ is the measured ellipticity in millidegrees, l is the cell path length, c is the protein concentration in grams per liter, and n = 303 residues. Deconvolution of the CD spectra into pure component spectra was performed using the algorithm CDSSTR [48] accessed through Dichroweb [49]. α-helical changes were followed observing the variation in MRE at 222 nm for the temperature stability measurements between 20°C and 95°C.

Fluorescence spectra were collected in an ISS Chronos fluorometer (ISS Champaign, IL) using a 280 nm diode as excitation source.
