# Correction: Structure and Stability of the Spinach Aquaporin SoPIP2;1 in Detergent Micelles and Lipid Membranes

**DOI:** 10.1371/annotation/589616b0-91e1-406c-8cf9-fe78ac08b8f6

**Published:** 2011-02-25

**Authors:** Inés Plasencia, Sabeen Survery, Sania Ibragimova, Jesper S. Hansen, Per Kjellbom, Claus Helix-Nielsen, Urban Johanson, Ole G. Mouritsen

Reference 24 was incomplete. The complete reference is: Nielsen CH (2009) Biomimetic membranes for sensor and separation applications. Anal Bioanal Chem 395: 697-718.

